# Mr. John Crichton

**Published:** 1860-11

**Authors:** 


MR. JOHN CRICHTON, who had attained great fame as a successful lithotomist, died at Dundee, Scotland, on the fourth of July, in the eighty-eighth year of his age.

